# Increase in foreign body and harmful substance ingestion and associated complications in children: a retrospective study of 1199 cases from 2005 to 2017

**DOI:** 10.1186/s12887-020-02444-8

**Published:** 2020-12-18

**Authors:** Arne Jorma Speidel, Lena Wölfle, Benjamin Mayer, Carsten Posovszky

**Affiliations:** 1 Department of Pediatrics and Adolescent Medicine, University Medical Center Ulm, Eythstr. 24, 89075 Ulm, Germany; 2 Institute of Epidemiology and Medical Biometry, Ulm University, Ulm, Germany

**Keywords:** Foreign body ingestion, Food bolus impaction, Caustic ingestion, Children

## Abstract

**Background:**

Children with a history of caustic or foreign body ingestion (FBI) seem to be presenting more frequently to emergency departments. This study aims to elucidate the clinical presentation, diagnostic procedures, and complications associated with the ingestion of different object categories over a 13-year time period.

**Methods:**

A structured retrospective data analysis of patients who presented between January 2005 and December 2017 to the University Medical Centre Ulm was performed. Patients up to 17 years of age with food impaction or foreign body or harmful substance ingestion were included by selection of the corresponding International Statistical Classification of Diseases and Related Health Problems (ICD10-GM) codes. Descriptive statistics, parametric or non-parametric tests, and linear regression analysis were performed.

**Result:**

In total, 1199 patients were analysed; the mean age was 3.3 years (SD 3.12; range 7 days to 16 years), the male to female ratio was 1.15:1, and 194 (16.2%) were hospitalized. The number of patients seen annually increased from 66 in 2005 to 119 in 2017, with a rise in percentage of all emergency patients from 0.82% in 2010 to 1.34% in 2017. The majority of patients (*n* = 619) had no symptoms, and 244 out of 580 symptomatic patients complained of retching or vomiting. Most frequently, ingested objects were coins (18.8%). Radiopaque objects accounted for 47.6%, and sharp objects accounted for 10.5% of the ingested foreign bodies, both of which were significantly more often ingested by girls (*p < 0.001* for both). Button battery ingestion was recorded for 63 patients with a significant annual increase (*R*2 = 0.57; β = 0.753; *p = 0.003*). The annual rate of complications also increased significantly (*R*2 = 0.42; β = 0.647; *p = 0.017*).

**Conclusion:**

We found an alarming increase in the number of children who presented to our emergency department with FBI and associated complications. A standardized diagnostic and therapeutic approach may reduce and prevent serious complications. Further preventive measures within the home environment are needed to stop this trend.

## Background

Accidental caustic and foreign body ingestions (FBI) are frequently reported in children world-wide and affect up to 75% of children under 6 years of age, since infants evaluate objects by tasting and swallowing [1–7]. A variety of objects and toxic bodies are ingested, among which coins appear to be the most commonly ingested world-wide, followed by fish bones, toys, jewellery, button batteries, magnets, household items, household cleaning substances, caustic sodas, and many others, and food impaction is also reported frequently in this cohort [1, 2, 5, 8–12]. The initial assessment includes a careful history-taking of symptoms, timing of the presentation, the type of foreign body or chemical agent, and associated conditions. This assessment is complemented by a physical examination assessing patient status, vital signs, and airway evaluation and emergency conditions. Management depends on the individual presentation of the patient, his or her underlying oesophageal pathology, the ingested object or liquid and its localization as well as on the physician’s clinical experience [7, 9]. Most objects will pass through the gastrointestinal tract (GIT) spontaneously, especially if they have already reached the small intestine and even up to 50% of large foreign bodies (FBs) with a diameter over 30 mm may pass the GIT without intervention [13, 14]. In contrast, FBs lodged in the oesophagus or sharp and relatively large FBs will require an intervention. In such cases, rigid or flexible endoscopy are typically used, where Foley catheter extraction, oesophageal bougienage, McGill forceps and magnetic catheters are less frequently implemented [5, 6, 15, 16]. Button batteries, multiple magnets and sharp objects need to be removed immediately to avoid serious complications [5, 7, 17–22]. Caustic agent ingestion is associated with high morbidity depending on the extent of injury on initial endoscopic evaluation [3, 9]. Food bolus impaction in children and adolescents may be a clinical feature of an underlying eosinophilic oesophagitis and not only requires a therapeutic but also a diagnostic endoscopy [23]. Depending on the objects and agents involved, a variety of associated complications are possible, ranging from mucosal abrasions, lacerations, perforations, mediastinitis, peritonitis, and strictures to death [5, 24, 25]. Complications are more likely if the FB remains impacted for an extended period of time [5, 26]. In addition, unobserved ingestion of objects or chemical substances may delay and complicate diagnosis and treatment, as infants may not inform their parents that they have ingested something [26]. This study aims to elucidate the clinical presentation, diagnostic procedures, and complications associated with the ingestion of different object categories over a 13-year time period. In particular, we evaluated the effect of implementing a diagnostic and therapeutic standard algorithm on the percentage of hospitalizations and complications.

## Methods

We performed a structured retrospective review of medical records collected over 13 years (from January 2005 to December 2017) from paediatric patients who presented with food bolus impaction and ingestion of foreign objects or chemical substances, at the Department of Paediatric and Adolescent Medicine, University Medical Centre Ulm, Germany. It is the only paediatric hospital of a catchment area in Germany that counts approximately 108,000 children. This study adheres to the ethical principles of the Declaration of Helsinki. It was approved by the Ethics Committee of Ulm University (No.399/17). For the presented data, the requirement to obtain informed consent was waived.

**Fig. 1:**
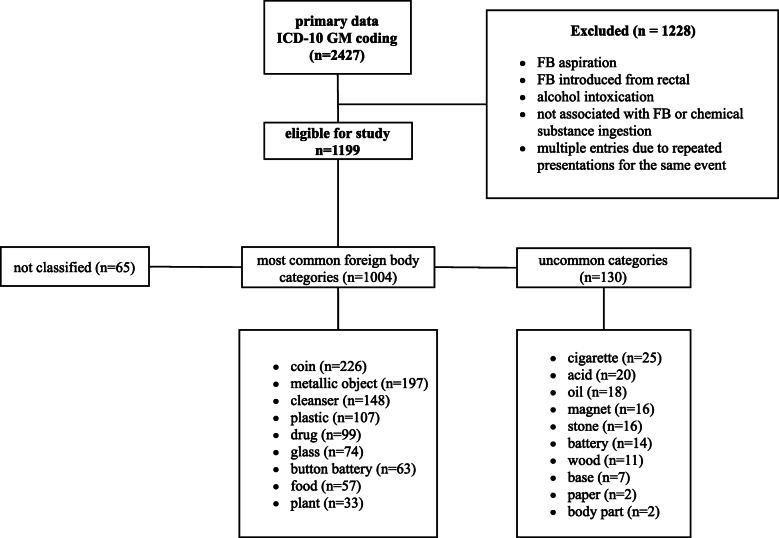
Flowchart depicting the study selection criteria

In total, 2427 records of patients aged 0 to 17 years were extracted from the hospital database using codes corresponding to FBI, chemical agents and food impaction from the International Statistical Classification of Diseases and Related Health Problems (ICD10-GM) codes including K22.1–3, T18.0–5, T28.5–9, T30.4, T52.0, T55, T60.8, T62.1–2, and T65.8–9 (Fig. 1). Episodes not corresponding to an accidental ingestion, e.g., alcoholic intoxication, iatrogenic injuries, and erroneous coding, were excluded and multiple presentations for the same event were merged.

Demographic patient information, associate conditions, type of foreign body or chemical substance, clinical symptoms, diagnostic and therapeutic management, and complications were collected from the electronic patient file and electronic database. The miscellaneous ingested foreign bodies, food boli and chemical substances were classified into 30 categories according to their characteristics (object type (solid, liquid and others), object form and size (below or over 2 cm*3 cm for infants, and below or over 3 cm*5 cm for children > 1 year of age, and sharp) [27], organic or inorganic, radiopacity, and object class (metal, plastic, wood, glass, food, drug, button battery, other batteries, coins, magnet, caustic solution, acid solution, oil, paper, plants, body parts, surfactant/cleanser, stone, and cigarette).

All data were entered and arranged in a single database spreadsheet (Excel XP, Microsoft Corporation, USA). Categorical data are reported as counts and percentages. Continuous data are reported as the mean and standard deviation or the median and minimum and maximum or range, according to data distribution. Further statistical analysis was performed using commercial statistical software (Statistical Package for Social Science (SPSS), version 26, IBM, Germany). To evaluate parametric data with independent samples, Student’s t-test was used. An inductive statistical evaluation of the nominal data was carried out using the chi-square test. This enabled the odds ratio to be calculated in suitable cases. Linear relationships between observed data over time were analysed by a linear regression model, and the percentage of variance in the dependent variable was expressed by the R-squared value. An explorative *p*-value < 0.05 was considered statistically significant.

All authors had access to the study data and reviewed and approved the final manuscript.

## Results

### Demographic data and longitudinal trends

In total, 1199 patients were evaluated. Most (*n* = 1102; 91.9%) presented to the hospital within the first 24 h after FB ingestion. The mean age was 3.3 years (median = 2.23, SD = 3.12; range = 16.97), with no significant variation in time (Table 1, Supplemental Fig. 1). Among the patients, 641 (53,3%) were male and 558 (46.5%) were female, with a ratio of 1.15:1 and a similar age distribution (mean age 3.17 and 3.45 years, respectively).

**Table 1:**
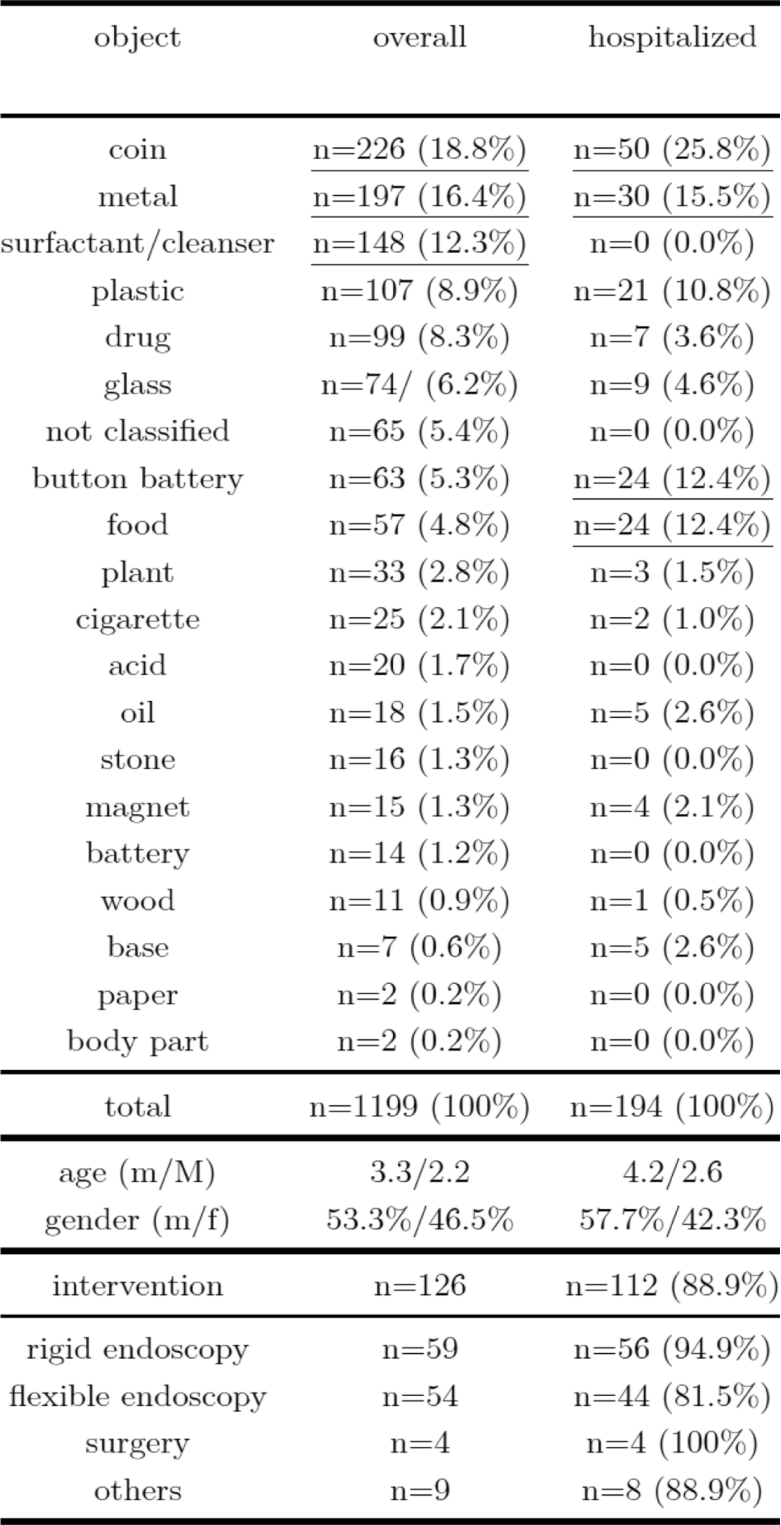
Patient characteristics, interventions, and distribution patterns of ingested objects, chemical substances and food

**Fig. 2:**
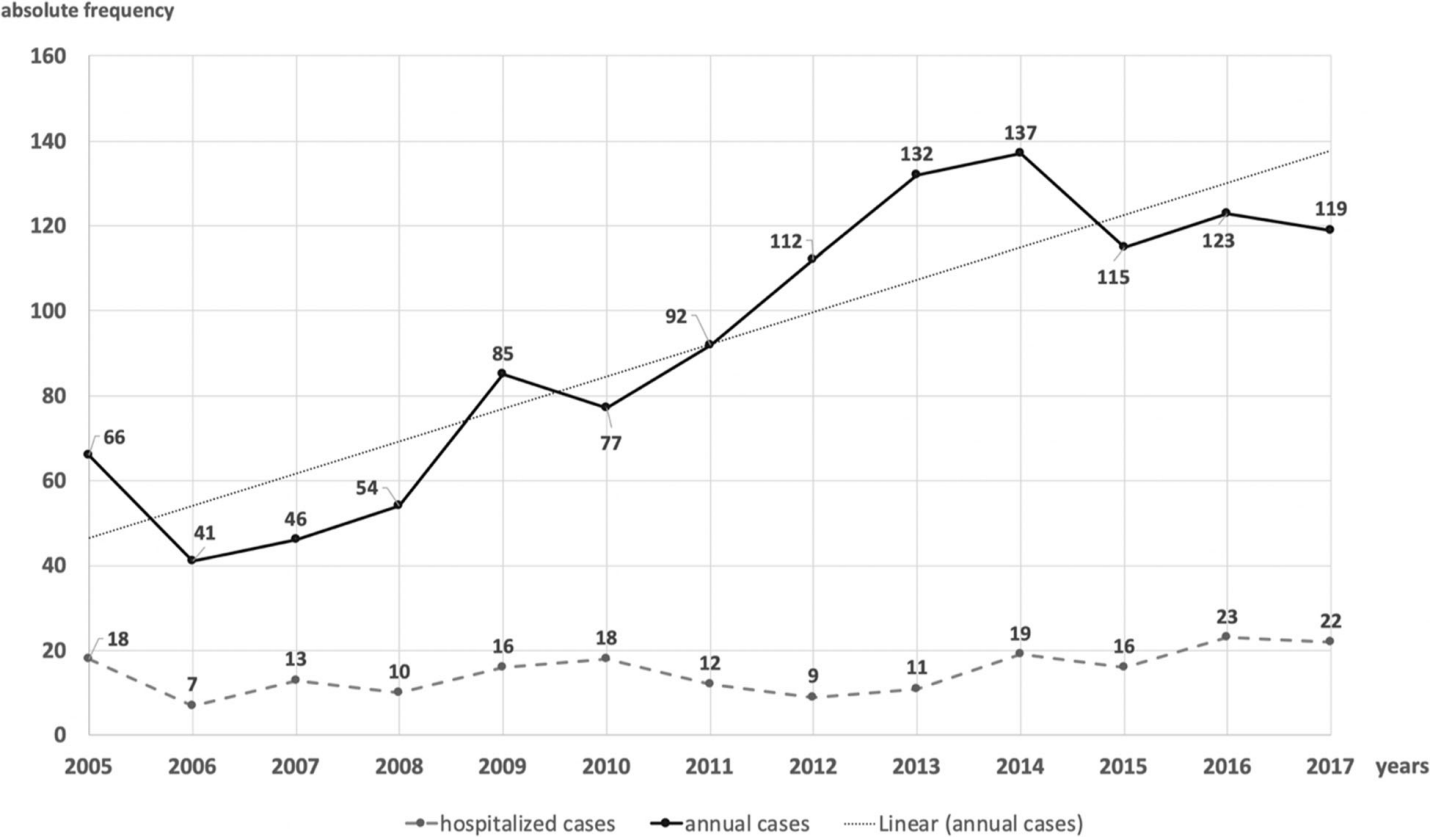
Annual number of children (black line) presenting with food bolus impaction and ingestion of foreign bodies or chemical substances to the Emergency Department from 2005 to 2017 and number of hospitalized patients (dashed grey line). Linear regression analysis (fine dotted grey line) revealed an R squared of 0.79 (β = 0.886; *p < 0.001*)

Annually, 41 to 137 patients were assessed, with a significant increase over time (*R*2 = 0.79; β = 0,886; *p < 0.001*) (Fig. 2). The annual rate increased by 80% from 6.1 in 2005 to 11.1 per 10,000 children in 2017 in the catchment area. This was also reflected by a rise in the percentage of all emergency cases from 0.82% in 2010 to 1.34% in 2017 (Supplemental Fig. 2). We noticed an annual increase in dangerous ingestions (e.g., sharp objects, magnets, batteries, caustic agents) during the observation period, which was accompanied by an absolute rise in FBI and thus did not affect the proportion (*R*2 = 0.11; β = − 0,336; *p = 0.262*). Dangerous objects were more often ingested by girls (52.5%).

Comorbidities were documented in 127 patients (10.6%). Psychiatric disorders (*n* = 16), intellectual disability (*n* = 13), postsurgical oesophageal atresia (*n* = 7), eosinophilic oesophagitis (*n* = 2) or other conditions (*n* = 101) were registered. Among the psychiatric disorders, five were diagnosed with emotional unstable personality disorders (F60.3), four with attention-deficit/hyperactivity disorders (F90.1), two with posttraumatic stress disorders (F43.1), two with atypical anorexia nervosa (F50.1), two with moderate and severe depressive episodes (F32.1/.2), and one with tobacco-induced mental health problems (F17.1). Most of them were already under psychiatric treatment at the time of ingestion. Food bolus impaction was associated with a higher rate of comorbidities (21.1%).

### Type of ingested objects/liquids and symptoms

Solid foreign bodies were specified in 921 patients, soft or flexible bodies in 67 patients (e.g. sponge, leaf), and liquids in 151 patients. For the remaining 60 (5%) patients, the ingested object was not specified. The majority of swallowed objects were inorganic objects (1018; 84.9%). Many objects were referred to as radiopaque FBs (*n* = 571; 47.6%), which were predominantly ingested by girls (*n* = 305; *p < 0.001*). Similarly, sharp objects (*n* = 126; 10.5%) were frequently swallowed by girls (*n* = 81; *p < 0.001*). Coins (*n* = 226), metallic objects (*n* = 197) and cleaning agents (*n* = 148) were the most common types of accidentally swallowed objects and liquids (Table 1). Button battery ingestion was recorded for 63 patients, presented with a significant annual increase (*R*2 = 0.57; β = 0,753; *p = 0.003*) and affected mostly toddlers, who were significantly younger (mean age 2.79 years; median 1.87; SD 2.40; range 11.64) than those who ingested coins (*p < 0.05*; effect size 0.214) (Table 3). Children who swallowed cleansers were significantly younger (mean age 2.5 years) than those who ingested coins, metals, plastics or drugs (*p < 0.05*).

Symptoms were documented in 619 patients (51.6%), mostly retching and vomiting (*n* = 244), followed by coughing fits (*n* = 152), pain (*n* = 133), hypersalivation (*n* = 44), dysphagia (*n* = 30) and miscellaneous complaints (e.g., abnormalities of behaviour, respiratory problems, globus sensation, minor bleeding) (Table 2). Medical complaints were significantly less frequent if the FB was localized distal to the flexura duodenojejunalis (odds ratio 0.44; 95% CI 0.29–0.68, *p < 0.05*) (Table 2).

**Table 2:**
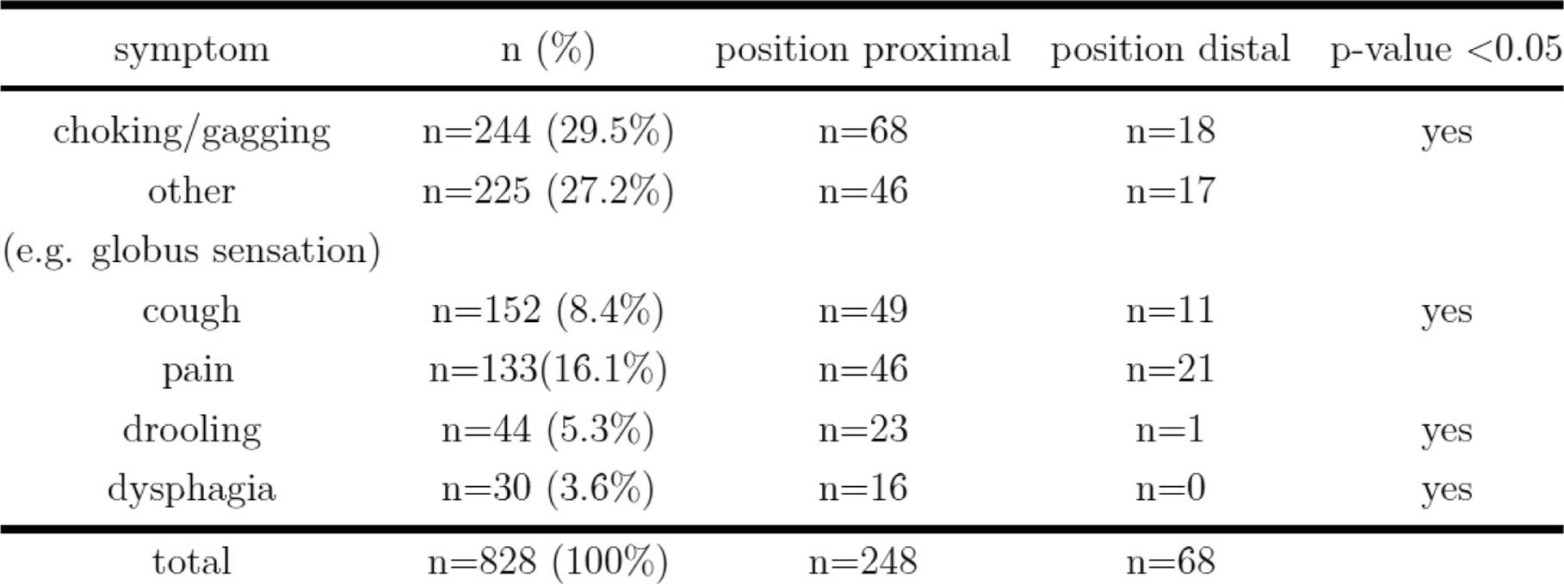
Distribution pattern of all registered symptoms according to the localization of the FB

Medical imaging was performed for 50.3% of the patients (*n* = 603) to localize the FB, and a significant rise in the annual examination rate was observed (*R*2 = 0.79; β = 0,886; *p < 0.001*). For girls, imaging was performed significantly more often (*n* = 313; *p < 0.001*). The main methods used were X-ray examination of the chest (*n* = 452; girls *n* = 243) and abdomen (*n* = 186; girls *n* = 90), followed by oesophagus contrast roentgenoscopy for 29 patients and other modalities (*n* = 33), including magnet resonance imaging (MRI), computer tomography (CT), or ultrasound. For 393 patients, the FBs could be clearly determined, 260 were located proximal (thereof 12 without symptoms) and 133 distal of the flexura duodenojejunalis (thereof 65 without symptoms). FBs were typically localized by X-ray (*n* = 350; 86.6%).

An oesophageal position for the FB was frequently associated with symptoms (83.9%). Most patients with food bolus impaction presented with symptoms (93%). The object-associated symptoms and complications are summarized in Table 3.

**Table 3:**
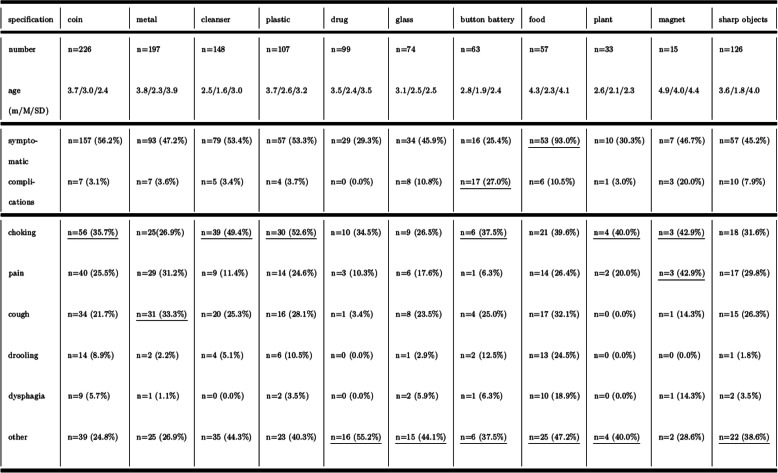
Object-associated symptoms and complications

### Management, complications and hospitalization

The primary management for the most FBIs was awaiting spontaneous passage, reassurance to the parents and guidance to perform faecal follow-up observations. Depending on the patient status, symptoms, and localization of the FB and suspicion of its fixedness, sharpness or harmfulness, FB therapeutic interventions were required. Interventional removal was performed for 126 patients, including rigid oesophagoscopy (*n* = 59) and flexible oesophagogastroduodenoscopy (*n* = 54), others tools (e.g. colonoscopy, McGill forceps) (*n* = 9) or surgery (*n* = 4) (Table 1). Successful recovery of the FB was documented in 128 patients, including 49 by flexible upper endoscopy (90.7%), 48 by rigid oesophagoscopy (81.3%), 4 by surgery, and 27 miscellaneous retrievals (e.g. colonoscopy, manual oral or rectal recovery, vomiting). The surgeries were performed due to oesophageal impaction of a button battery with fistulation, multiple magnets leading to bowel perforation and fever, bowel obstruction by a chestnut with obstructive ileus, and a pistachio shell that migrated through the wall of the oesophagus into the mediastinum causing stridor.

Complications were retrieved for 65 patients (5.4%), and a significant rise in the annual rate was observed (*R*2 = 0.42; β = 0,647; *p = 0.001*). Most patients had mucosal injuries of various degrees, frequently superficial (*n* = 56), followed by deeper caustic tissue damage (*n* = 11), necrosis (*n* = 10), and perforations (*n* = 5) but no fistulations. Fever (*n* = 10) and other complications (*n* = 12) were observed, but no deaths occurred. The percentage of complications with respect to all registered patients dropped by 2 points or 29% from 6.8% in the years 2005–2010 to 4.8% in the years 2011–2017 after implementation of a diagnostic and therapeutic algorithm at our hospital.

Simultaneously, the percentage of hospitalized patients dropped by 4.7 points or 23% from 20.6% in the years before to 15.9% after application of this algorithm, while the length of the hospital stay was equivalent (mean 2.98/SD 4.0 versus 3.01 SD 2.9).

Admission to the hospital was necessary for 194 (16.2%) patients, ranging from 7 to 22 per year (mean = 14.9) (Fig. 2), with a mean duration of hospitalization of 2.99 days (median = 2.0; SD = 3.41; range 1–27 days). Hospital admission was relatively more frequent after food bolus impaction (42.1%) and button battery ingestion (38.1%), with 24 patients each (Table 1).

## Discussion

The analysis of 1199 accidental paediatric foreign body and chemical substance ingestions over 13 years (2005–2017) at a German University Medical Centre revealed a significant annual increase of 80% from 6.1 in 2005 to approximately 11 per 10,000 children in 2017 in the catchment area.

Overall, the annual rate of complications also significantly increased, which was probably restricted by the implementation of a diagnostic and therapeutic algorithm at the end of 2010.

Ingestion of foreign bodies or chemical substances is frequent among children below 6 years of age around the world [1–5]. The majority of patients who presented to our hospital were toddlers (median 2.2 years) and predominantly male (53.3%). In addition, we registered comorbidities such as psychiatric disorders, intellectual disability, postsurgical oesophageal atresia and eosinophilic oesophagitis which may also predispose patients to FBI or food bolus impaction [28, 29].

A variety of foreign bodies were ingested, of which coins were the most common in our cohort, as in many other studies [1, 5, 8, 11, 12]. However, the proportion was lower in our study (18%) than that reported by others (49 to 88%), which may reflect changes over time or differences in patient referral, selection and inclusion, in object classification and localization, or in the diets and habits of the patient populations [4, 5, 12, 30, 31] (supplemental Table). The ingestion patterns also differed by sex, age and season (e.g., Christmas decorations) [8, 32]. We found, that girls were at risk for harmful ingestion of sharp objects in our population. Another study showed that it is 2.5 times more likely that girls rather than boys ingest jewellery or hair products compared to boys [8]. We also found relevant liquid ingestions of potentially harmful cleansers (12.3%), acids (2.7%) or bases (0.6%). Caustic ingestions in children are mostly accidental, and the severity depends on the type and quantity of the ingested substance [33].

We observed an alarming trend concerning the number of accidental ingestions in our population. This has also been reported in the United States, with an annual increase of 91.5%, from 9.5 in 1995 to 18 per 10,000 children with FBI in 2015 [8]. In contrast, considerably fewer FBIs have been reported at Chiang Mai University in Thailand (only 194 cases from 2006 to 2017), with a population comparable to Ulm. This cannot be explained by different age limits (< 15 versus < 18 years) and the inclusion of ingestion of chemical substances in our study. Thus, other factors, e.g., difficult access to medical care or fewer harmful items in the household, may influence the observed lower frequency of FBIs. The increase in our study affected all categories of objects and substances, many of which are increasingly used in German households. In particular, we observed an alarming increase in lithium button battery ingestions in infants, which was associated with hospitalisation and major complications. This trend had also been previously reported in comparable studies from the U.S. [34, 35].

In our cohort, the presenting symptoms varied depending on the type and localization of the ingested FB; e.g., gagging, pain and coughing were frequently observed after coin ingestion. In contrast, a primary association with vomiting and drooling had been reported in other studies after coin ingestion [5]. Patients with oesophageal FB mainly present with drooling, vomiting and dysphagia, especially if the FB was located in the first narrowing of the oesophagus [36]. Nevertheless, most patients had a normal physical examination [31]. In children, the diagnosis of FB ingestion may be complicated if the ingestion is not observed or the child is asymptomatic. As in other studies, approximately half of the patients were asymptomatic [2].

A carefully obtained history, the type of ingestion and the level of suspicion will determine the course of action to avoid severe and life-threatening complications. In this study, approximately 16% of all patients required hospitalization, which is slightly more than in similar studies that have reporting approximately 10% [8]. Twenty-two percent of patients who ingested coins and more girls than boys were hospitalized. In fact, the hospitalization rate for coin ingestions was considerably lower in our cohort than the 55% reported in a European study [4]. The highest rate of complications was observed for button battery, glass and food ingestions. Others have reported an increased prevalence of complications after sharp FB ingestion and a 4- to 8-fold increase in complications if the endoscopic retrieval of the oesophageal FB was performed beyond 24 to 48 h after ingestion [37]. Serious complications and fatal outcomes have been reported for button battery ingestions [18, 19, 25]. Although we observed an annual increase in total patients and harmful ingestions, the number of patients admitted to the hospital remained constant.

In 2010, we implemented an algorithm for the diagnostic and therapeutic management of FBI at our hospital that takes into account the age of the patient, symptoms, and the size, type and location of the radiopaque and radiolucent FB based on an interdisciplinary consensus from the departments of radiology, paediatric and adolescent medicine (including paediatric intensive care and paediatric gastroenterology), and ENT [27]. This may potentially influence the practice of admissions at our hospital and partially explain the constant number of hospitalized patients. In addition, the complication rate was reduced by 29%.

The European Society of Paediatric Gastroenterology and Nutrition (ESPGHAN) and European Society of Gastrointestinal Endoscopy (ESGE) recommend X-ray examination for all patients with suspected FB ingestion even without symptoms [22]. In our study, medical imaging was performed for half of the patients, as nearly half of the objects were radiopaque. Imaging is important to confirm the presence, type, number and localization of FBs as well as to detect complications and guide further management and follow-up if indicated [38]. X-ray of the chest and abdomen was frequently performed and detected and localized the FB in 86% of the patients. In fact, we observed an annual increase in the number of detected FBs, particularly through X-ray investigations. Although hand-held metal detectors (HHMD) have been shown to detect and localize the majority of metallic FBs, we did not use them in our department at that time [39]. It has been shown that HHMDs have a high sensitivity in the detection of coins and seems to be a good early screening tool for faster triage in the emergency room setting, potentially reducing radiation exposure [40, 41].

The primary management was awaiting spontaneous passage for most FBIs, and interventional removal was performed for 126 patients. The indication and timing of medical intervention to remove a foreign body was based on the location, size and type of the FB, the duration of impaction, and patient symptoms according to our in-house standard with special attention towards button battery and magnet ingestions [27]. The initial localization and size of the FB are determining factors of the likelihood of spontaneous passage [5, 13].

Removal of the FB was mainly performed by rigid or flexible endoscopy according to the localization of the FB and symptoms of the patient and was successful in 81% of rigid and 90% of flexible endoscopy. High success rates for rigid and flexible endoscopy have been reported in retrospective studies on oesophageal FBs among children and adults with low rates of complications (supplemental Table) [15, 36, 42, 43]. The management of foreign body, food and toxic substance ingestions needs to be adapted for infants, e.g., smaller endoscopes in children below ten kilos, and paediatric-trained endoscopists are required [22, 44]. Oesophageal food impaction is a frequent finding and requires special attention [45]. Removal is performed either en bloc or by a piecemeal approach using various grasping devices and after examining the oesophagus distal to the bolus; the push technique is also sometimes used [20]. Food impaction is frequently associated with oesophageal pathology, e.g., postsurgical oesophageal atresia and eosinophilic oesophagitis [28, 46, 47]. Magnet ingestion is rarely registered but frequently requires surgery, especially for children with neurological or psychiatric diseases who have an increased risk of ingesting multiple magnets [48, 49]. There has been an alarming increase in magnet ingestions in emergency departments according to data from the National Electronic Injury Surveillance System (NEISS) [21].

We found a very low need to perform surgical interventions (0.3% of all, 2% of hospitalized cases, 3% of removed FB) in our cohort, unlike the 18% reported by others for hospitalized patients, despite providing paediatric surgery twenty-four-seven [50]. Cohorts that once required surgical interventions could be treated by experienced endoscopic removal today [26]. In children, mild oesophageal lesions (88%) have been predominantly identified following caustic ingestions, and severe oesophageal lesions have been associated with the presence of signs and symptoms (e.g., oral lesions, vomiting, dyspnoea, drooling, dysphagia, and haematemesis) [51]. Thus, endoscopy could be avoided in the absence of signs and symptoms [51], as in our study cohort.

In our study, 9% of the children presented to our hospital more than 24 h after ingestion versus the 22% reported in a study from Pittsburgh on children who underwent oesophagoscopy for suspected FB [31]. The lack of awareness of harmful situations requiring emergency care and prompt intervention may lead to serious complications, e.g., as observed with button batteries, pins and magnets [4]. Preventive measures, e.g., pressure on manufacturers to package items appropriately for children below 3 years of age, effectively reduced toy ingestions in the U.S. in 2011 [8]. Furthermore, food and toys should not be marketed together to prevent children from ingesting toys. Legal measures to protect children from the growing problem of unintentional button battery ingestion are required and should include child-resistant packaging for batteries, child-resistant closures for all consumer products that use button batteries, and warnings regarding the potential danger of ingestion [52, 53].

Finally, few limitations of this study should be underlined. First, as we incorporated a retrospective study design, the conditions may not be ideal and lack some relevant information not available from the electronic patient file. In addition, we only captured information from patients who presented to our hospital, which, as in many other studies, underestimates the real frequency of accidental ingestions among children. Additionally, comparability among related studies is limited, as patient selection and categorization of objects may differ, and age groups may vary [5].

## Conclusion

This study shows a significant increase in ingested household items and liquids associated with complications over 13 years in the studied population.

Appropriate care for children with FBI requires proper management, skilled physicians, adequate equipment and, if necessary, inpatient admission. The implementation of clear diagnostic and therapeutic algorithms in paediatric emergency departments may lower the progression of injuries and complications prospectively.

Additional preventive measures, including public health interventions and superior consumer safety standards, are necessary to stop the observed trend and should particularly focus on the preschool toddler group.

## Supplementary Information

**Additional file 1: Supplemental Figure 1.** Annual age distribution from 2005 to 2017 of the 1199 children presenting with food bolus impaction or ingestion of foreign bodies or chemical substances to the Department of Paediatrics and adolescent Medicine, University Medical Centre Ulm, Germany. Annual mean age (black line) and minimal (dashed black line) and maximum age (dashed grey line) are indicated.

**Additional file 2: Supplemental Figure 2.** Annual percentage of patients (black line) presenting with food bolus impaction or ingestion of foreign bodies or chemical substances out of all patients presented to the Emergency Department from 2009 to 2017. Linear regression analysis (grey dotted line) revealed a significant increase in this percentage over time (*R*2 = 0.83; β = 0.912; *p = 0.001*).

**Additional file 3: Supplemental Table.** Worldwide distribution pattern of ingested foreign bodies and their management.

## Data Availability

The datasets and analysis used during the current study are available from the corresponding author on reasonable request.

## Abbreviations

ENT: Ear, nose, throat
FB: Foreign body
FBI: Foreign body ingestion
