# Supplementary material for: Increase in foreign body and harmful substance ingestion and associated complications in children: a retrospective study of 1199 cases from 2005 to 2017
Source: BMC Pediatr. 2020 Dec 18;20:560. doi: 10.1186/s12887-020-02444-8 (PMC7747382; doi:10.1186/s12887-020-02444-8)
Supplement: Supplementary file 1 — Additional file 1: Supplemental Figure 1. Annual age distribution from 2005 to 2017 of the 1199 children presenting with food bolus impaction or ingestion of foreign bodies or chemical substances to the Department of Paediatrics and adolescent Medicine, University Medical Centre Ulm, Germany. Annual mean age (black line) and minimal (dashed black line) and maximum age (dashed grey line) are indicated. [file 12887_2020_2444_MOESM1_ESM.pdf]

Supplemental figure 1

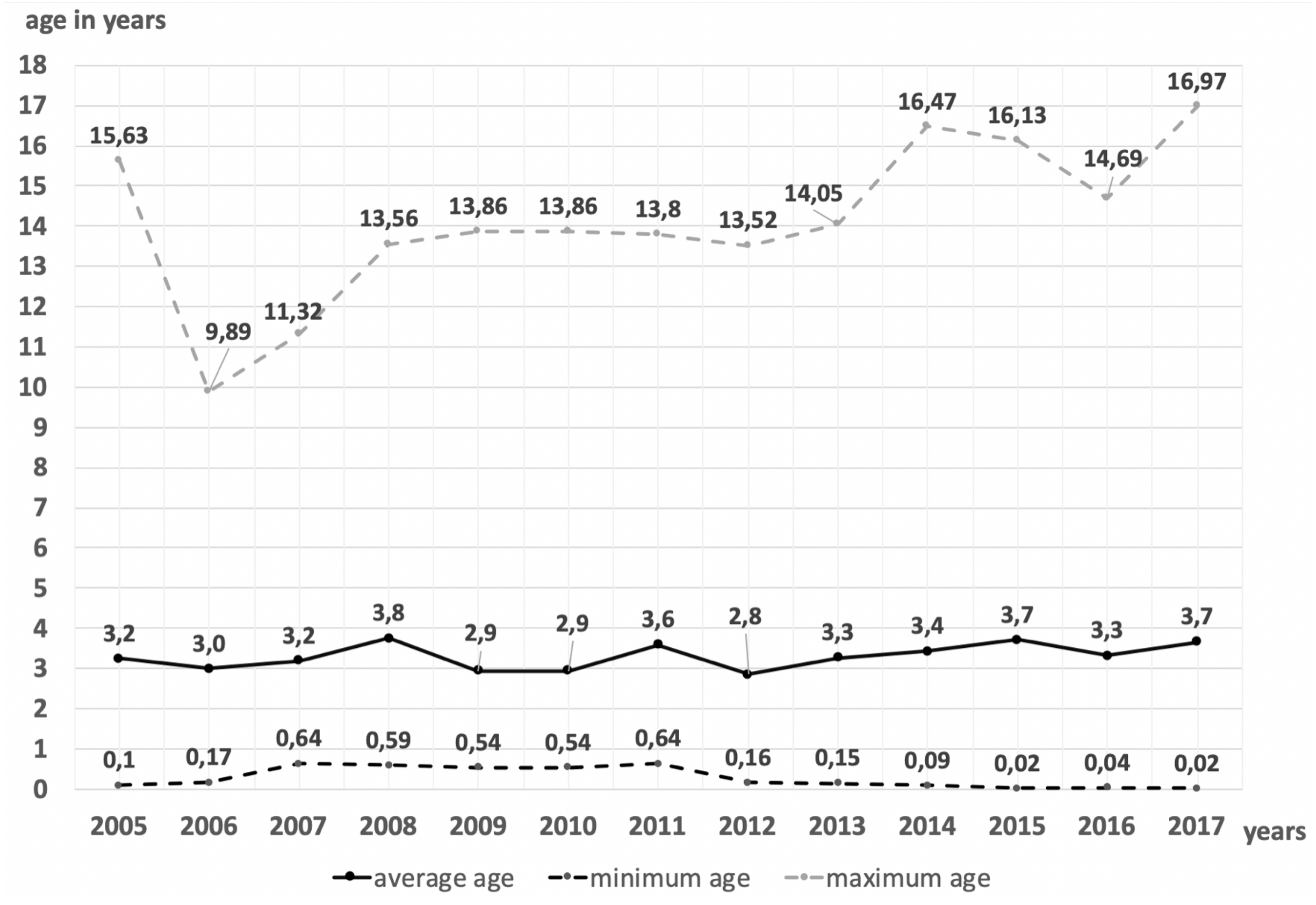

Annual age distribution from 2005 to 2017 of the 1199 children presenting with food bolus impaction, ingestion of foreign bodies or chemical substances at the Department of Pediatrics and adolescent Medicine, University Medical Center Ulm, Germany. Annual mean age (black line), minimal (dashed black line) and maximum age (dashed grey line) are indicated.
