# Supplementary material for: Increase in foreign body and harmful substance ingestion and associated complications in children: a retrospective study of 1199 cases from 2005 to 2017
Source: BMC Pediatr. 2020 Dec 18;20:560. doi: 10.1186/s12887-020-02444-8 (PMC7747382; doi:10.1186/s12887-020-02444-8)
Supplement: Supplementary file 2 — Additional file 2: Supplemental Figure 2. Annual percentage of patients (black line) presenting with food bolus impaction or ingestion of foreign bodies or chemical substances out of all patients presented to the Emergency Department from 2009 to 2017. Linear regression analysis (grey dotted line) revealed a significant increase in this percentage over time (R2 = 0.83; β = 0.912; p = 0.001). [file 12887_2020_2444_MOESM2_ESM.pdf]

Supplemental figure 2

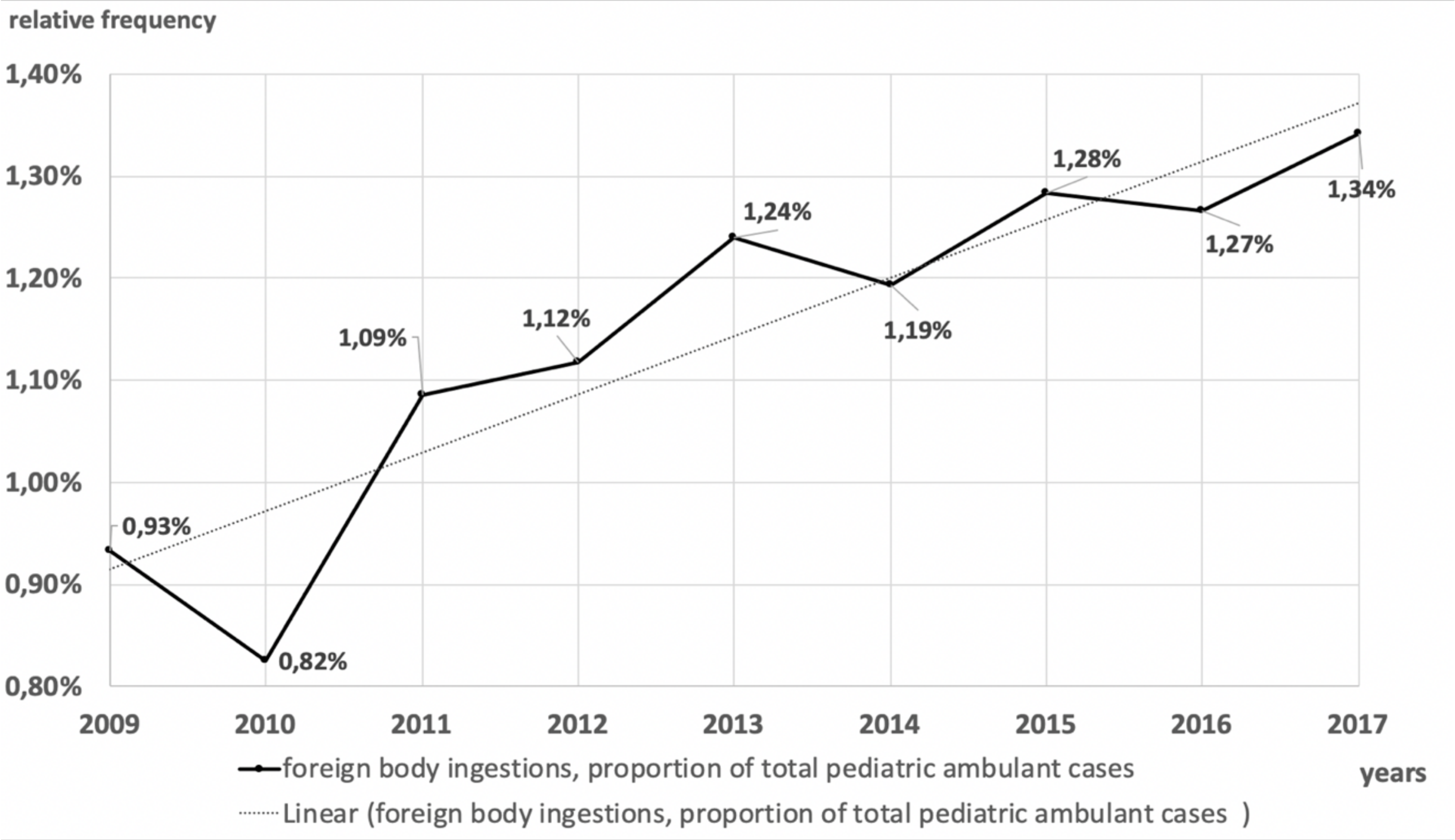

Annual percentage of cases (black line) presenting with food bolus impaction, ingestion of foreign bodies or chemical substances of all cases presented at the Emergency Department from 2009 to 2017. Linear regression analysis (grey dotted line) revealed a significant raise of this percentage over time ($R2=0.83$; beta=0,912; $p= 0.001$).
