# Supplementary material for: Increase in foreign body and harmful substance ingestion and associated complications in children: a retrospective study of 1199 cases from 2005 to 2017
Source: BMC Pediatr. 2020 Dec 18;20:560. doi: 10.1186/s12887-020-02444-8 (PMC7747382; doi:10.1186/s12887-020-02444-8)
Supplement: Supplementary file 3 — Additional file 3: Supplemental Table. Worldwide distribution pattern of ingested foreign bodies and their management. [file 12887_2020_2444_MOESM3_ESM.docx]

**Supplemental table: Worldwide distribution pattern of ingested foreign bodies and their management**

| Author, year | Department | Country  City | Study type,  Period, | Sample size | Age (years)  (mean/SD/  range) | Gender  boy:girl | Commonest foreign body (%) | Management (n) |
| --- | --- | --- | --- | --- | --- | --- | --- | --- |
| Cheng & Tam,  1999 | Paediatric surgery Emergency department | China,  Hong Kong | Retrospective case series  1964-1997  Patients ≤16 y | 1265  552 proven | 5.2/-/0-16 | 1.27:1 | Coins (49)  Fish bones (29)  Metallic objects (13) | Direct laryngoscopy (73)  FE (44)  RE (32) |
| Wai Pak et al., 2001 | Otolaryngology Emergency department | China,  Hong Kong | Prospective cohort  1993-1996 | 311  115 proven | 5.18/4.1/0-12 | 1.39:1 | Fish bones (88.7)  Chicken bones (3.2) Coins (2.4) | Direct vision, indirect or Mackintosh laryngoscopy,  Flexible laryngoscopy, Rigid esophagoscopy |
| Khorana et al.,  2019 | Paediatric surgery  Paediatric gastroenterology  otolaryngology | Thailand,  Chiang Mai | Retrospective case series  2006-2017  Patients <15 y | 194 | Median 3.6  Range 1-6 | 1.15:1 | Coin (41.2)  Food bolus (15.5)  Button battery (10.8) | Endoscopy (69)  Surgery (2)  Others (6) |
| Chotigavanich et al., 2012 |  | Thailand, Bangkok | Prospective cohort  2006-2010 | 172  10 in airways |  | 1.38:1 | Bone (38.8)  Denture (11.6)  Other food (9.9) |  |
|  | ESFBI | Finnland | Case series | 307  18 in airways |  | 1.45:1 | Bone (15.6)  Pearl, ball, marble (13.3)  Toy (13.3) |  |
|  | ESFBI | Slovenia | Case series | 104  2 in airways |  | 1.41:1 | Pearl, ball, marble (15.4)  Nut. Seed. Beans (13.5)  Bone (12.5) |  |
|  | ESFBI | Sweden | Case series | 235  6 in airways |  | 1:1 | Pearl, ball, marble (35.3)  Nut. Seed. Beans (14.5)  Pebble (7.7) |  |
| Yalcin et al.,  2007 | Paediatric surgery | Turkey  Ankara | Retrospective case series  1973-2005 of hospitalised patients | 112 | 2.27/2.84 | 1.09:1 | Safety pin (41)  Coins (22.3) | Laryngoscopy with McGill forceps (10),  RE (43)  FE (4)  Surgery (12) |
| Arana et al., 2001 | Paediatric gastroenterology  Pediatric Emergency deparmtent | Belgium,  Brussels | Retrospective case series  1985-2000 | 325 | 2.8/-/0-18 | 1.38:1 | Coins (27)  Sharp objects (16)  Batteries (13)  Toy parts (12)  Large food bolus (12) | McGill forceps (28)  Endoscopy (81) |
| Speidel et al.,  2020 | Paediatric Emergency Dep.  Paediatric Gastroenterology  Paediatric Surgery  ENT Dep. | Germany,  Ulm | Retrospective case series  2005-2017 | 1199 | 3.3/3.12/0-16 | 1:15:1 | Coin (18.8)  Metallic object (16.4)  Chemicals (14.6) | RE (126)  FE (54)  Others e.g. McGill forceps (9) |
| Leskova et al.,  2019 |  | Czech Republic Hradec Králové | Retrospective case series 2011-2018 | 365 | 2.4/-/- | 0.99:1 | Coins (26)  Batteries (14.5)  Sharp objects (5.2) | Endoscopy (63) |
| Orsagh-Yentis et al., 2019 | US emergency departments | U.S.A. | Retrospective  1995-2015 national electronic injury surveillance system for children <6 y | 29893 |  | 1.13:1 | Coins (61.7)  Toys (10.3)  Jewellery (7)  Batteries (6.8) |  |
| Panieri & Bass, 1995 | Trauma Unit and Department of paediatric surgery | South Africa  Cape Town | Retrospective case series  1991-1994 | 663 | -/-/0-13 | 1.35:1 | Coins (37.9)  Sharp objects (21.7)  Bones (13.4) | McGill Forceps (6)  RE (76)  Balloon catheter (27) |
| Fujisawa et al., 2020 | Paediatric Emergency  General Paediatrics | Japan  Itabashi | Retrospective case series  2013-2018  <16 y | 252 | Median 1.3  Range 0-15 | 1.25:1 | Cigarettes (17)  Plastics (17)  Chemicals (11) |  |
| Denney et al.,  2012 | Paediatric gastroenterology | U.S.A.  Mississippi | Retrospective case series  1998-2008  EGD for FBI-removal | 248 | 3.9/3.2/- | 1.6:1 | Coin (81)  Jewellery (4)  Battery (3.2)  Hardware (2.8) | FE (248) |
| Popel et al.,  2011 | Paediatric Gastroenterology  Paediatric Otolaryngology | Canada  Edmonton | Retrospective case series  2005-2008  oesophageal FB removal | 140 | 4.98/4.1/0-16 | 1.37:1 | Coins (77.9)  Food impaction (12.9) | FE (87)  RE (51) |
| Little et al., 2006 | Emergency Department  Paediatric Surgery | U.S.A.  Kansas City | Retrospective case series 1988-2004  Oesophageal FB | 555 | 3.24/-/0-19 | 1.15:1 | Coins (88) | Foley balloon extraction (468)  RE (59) |

ESFBI = European Survey on Foreign Bodies Injuries; EGD = esophagogastro-duodenoscopy; FE= flexible endoscopy; RE = rigid endoscopy
